# An Analysis on Groundwater Recharge by Mathematical Model in Inclined Porous Media

**DOI:** 10.1155/2014/189369

**Published:** 2014-08-26

**Authors:** Shreekant P. Pathak, Twinkle Singh

**Affiliations:** Department of Applied Mathematics & Humanities, S. V. National Institute of Technology, Surat 395007, India

## Abstract

The present paper discusses the analysis of solution of groundwater flow in inclined porous media. The problem related to groundwater flow in inclined aquifers is usually common in geotechnical and hydrogeology engineering activities. The governing partial differential equation of one-dimensional groundwater recharge problem has been formed by Dupuit's assumption. Three cases have been discussed with suitable boundary conditions and different slopes of impervious incline boundary. The numerical as well as graphical interpretation has been given and its coding is done in MATLAB.

## 1. Introduction

In nature, groundwater is a key element, in many geological and hydrochemical processes, which sustains spring discharge, river base flow, lakes, and wetlands. Groundwater, extracted from deep geological formations (called aquifers) through pumping wells, constitutes an important component of many water resource systems. Groundwater recharge problem has been discussed by many researchers with different viewpoints. Patel et al. [1] have obtained a series solution of moisture content in vertical groundwater flow through unsaturated heterogeneous porous media. Swaroop and Mehta [2] have obtained a solution to the problem of one-dimensional flow in unsaturated porous media taking finite element approach. Swartzendruber has used Philips [3] method to get graphical illustration of the mathematical solution for horizontal water function. Verma and Mishra [4] have obtained solution by similarity transformation of one-dimensional vertical ground water recharges through porous media. Mehta [5] has obtained an approximate solution by the method of singular perturbation technique. This paper is mathematically formulated by Dupuit's assumption. Dupuit [6] based his assumptions on the observation that, in most groundwater flows, the slope of the phreatic surface is very small. He assumes that, for the small inclinations of free surface, the velocity is proportional to the slope of free surface but independent of the depth. The proportionality coefficient *K* (hydraulic conductivity) is a property of vascular plants, soil, or rock that describes the ease with which water can move through pore spaces or fractures. There are different values of *K* according to different types of porous media; that is, for homogeneous porous media it is constant and for heterogeneous porous media it is the function of distance *x*. For this mathematical model we considered *K* = 10^−4^ cm/sec. If one of the boundaries of aquifer is flow line or flow surface, the flow analysis is difficult especially for the analytical solution. The water table is flow line or flow surface when there is no recharge from unsaturated zone. Water table is neither flow line nor equipotential line in case of having input flow from vadose zone, and it crosses flow lines according to Todd and Mays [7]. In the analytical method Dupuit's assumption [6] has been applied. Hantush and Cruz [8] have used (1) for flow of water on inclined impermeable boundary:

(1) q=−K(y−mx)dydx, where  m=tanθ.

Bear and Alexander [9] have presented the analytical solutions to the one-dimensional fluid flow and steady state on inclined boundary. They considered a constant value for flux *Q*_0_ for the above condition. Their solution also handled the infiltration from the unsaturated zone to water table.

## 2. Formation of Mathematical Model of the Groundwater Flow

**Figure 1:**
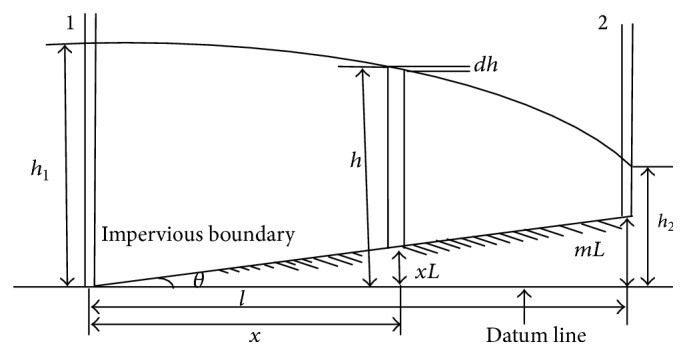
This figure represents unconfined aquifer on incline impervious boundary with positive slope.

In nature, groundwater is in very large land scale; to understand this problem, we form a mathematical model such that the homogenous porous media are laid on impervious inclined layer with *θ* angle as shown in Figure 1. If the channels 1 and 2 on boundaries are filled with water to heights *h*_1_ and *h*_2_, respectively, with respect to the datum line, then the system will reach steady state for such fluid flow problem.

If the flow rates in the direction of *x* and *y* are denoted by *q*_*x*_ and *q*_*y*_, respectively, in the mathematical model then the continuity equation can be written as

(2) ∂qx∂x+∂qy∂y=0.

But here we have considered only one-dimensional fluid flow in mathematical model (considering fluid flow in *x* direction only); then,

(3) ∂qx∂x=0⟹qx=c1.

By using Dupuit's assumption for the model we get

(4) −K(h−mx)dhdx=c1.

By rewriting the above equation,

(5) c1dx+K(h−mx)dh=0.

Equation (5) represents a mathematical model in which ordinary differential equation arises during one-dimensional groundwater recharge phenomena. It is an ordinary differential equation which is not exact; now to convert it into exact, we multiply (5) with integrating factor *e* ^(*Km*/*c*_1_)*h*^:

(6) c1e(Km/c1)hdx+Ke(Km/c1)h(h−mx)dh=0.

As per Figure 1, we choose appropriate boundary conditions:

(7) h(x=0)=h1,  h(x=l)=h2.

The solution of (6) is given by

(8) (mx−h+c1Km)e(Km/c1)h=c2.

To determine constants of integration *c*_1_ and *c*_2_ by using boundary conditions equation (7), we get

(9) c1=h2Km−Km2+(c1−Kmh1)e(Km/c1)(h1−h2),

(10) c2=(c1Km−h1)e(Km/c1)h1.

Three cases have been considered in analysis and consistency of the mathematical model and its solution with suitable boundary conditions and different slopes of impervious incline boundary.

**Case 1 .**

**Figure 2:**
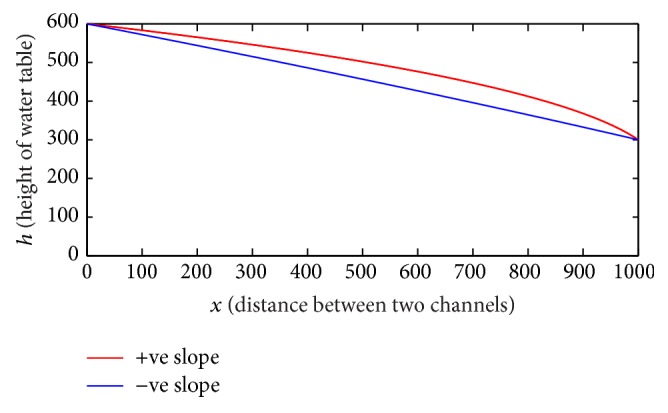
This figure represents the free surface of water table: *h*_1_ > *h*_2_ for *m* > 0 and *m* < 0.

In first case, let the total heads of channels 1 and 2 be 600 cm and 300 cm, respectively, for the mathematical model. The distance between two channels is *l* = 1000 cm and the hydraulic conductivity of the soil is *K* = 10^−4^ cm/sec. Now, if we take positive slope of impervious boundary *m* = 0.2, we can get constants and *c*_1_ = −0.00973 and *c*_2_ = −316.768. In the same way for negative slope *m* = −0.2 we can get *c*_1_ = −0.01659 and *c*_2_ = 473.082. After substituting the values of and in (8) we get the two curves of free surfaces for the top flow lines as shown in Figure 2, such that the upper curve represents free surface for positive slope *m* = 0.2 and the lower curve represents free surface of water for negative slope *m* = −0.2.

**Case 2 .**

**Figure 3:**
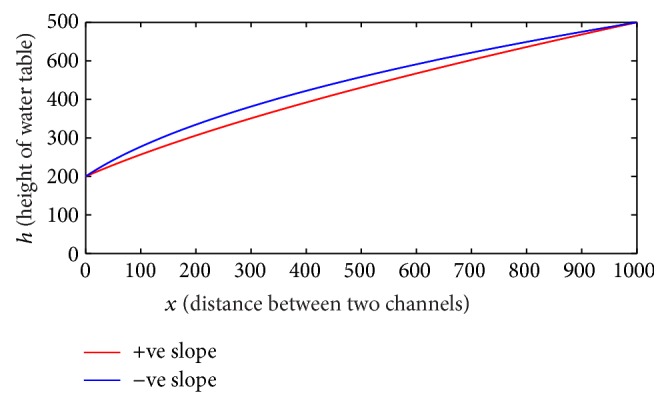
This figure represents the free surface of water table: *h*_1_ < *h*_2_ for *m* > 0 and *m* < 0.

In the second case, let the total heads of channels 1 and 2 be 200 cm and 600 cm, respectively, for this mathematical model. The other parameters are same as Case 1. Now for the positive slope we get *c*_1_ = 0.01242 and *c*_2_ = 581.305 and for negative slope *c*_1_ = 0.01917 and *c*_2_ = −940.203. Using these constants in (8) gives us the upper flow curves of free surface of water as shown in Figure 3, such that the lower curve represents free surface for positive slope *m* = 0.2 and the upper curve represents free surface of water for negative slope *m* = −0.2.

**Case 3 .**

This case is considered for the same pressure head of both channels 1 and 2 for this mathematical model. Therefore, *h*_2_ = *h*_1_ + *ml*. Substituting this value in (9) we get

(11) c1=Kmh1+(c1−Kmh1)e−(Km2l/c1).

The numerical value of *c*_1_ for positive slope *m* = 0.2 in (11) is 0.006, which is same as *Kmh*_1_; that is, *c*_1_ = *Kmh*_1_ = 0.006 and *c*_2_ = 0. Using the values of *c*_1_ and *c*_2_ in (8) we get

(12) h=mx+h1.

Equation (12) is an equation of straight line having *h* intercept as *h*_1_ with positive slope *m* = 0.2, and for negative slope *m* = −0.2, *c*_1_ = 0.00972 and *c*_2_ = −424.33.

**Figure 4:**
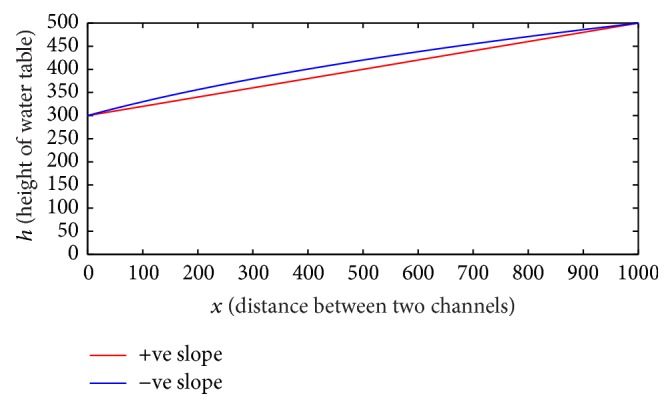
This figure represents the free surface of water table for equal pressure head and *m* > 0 and *m* < 0.

Figure 4 shows that the upper flow line represents free surface of water for positive slope and is parallel to the inclined impervious boundary.

## 3. Mathematical Analysis of Flow Rate

Differentiates (8) with respect to *x*, which gives change in free surface with respect to distance *x*. Hence,

(13) dhdx=m1−(c2Km/c1)e−(Km/c1)h.

Also, Darcy's law gives the flow rate of the system as

(14) q=−K(h−mx)dhdx,q=Km(mx−h)1−(c2Km/c1)e−(Km/c1)h.

Thus, (14) gives flow rate expressed in the terms of distance *x* and the negative exponential function of height *h* of free surface of water.

## 4. Numerical and Graphical Interpretation of Mathematical Model

In the first case, as distance *x* increases, height *h* decreases; it shows that the water level of groundwater decreases more speedily when impervious boundary has negative slope compared to positive slope of the impervious boundary, which is consistent physically as well as mathematically and also holds natural area for recharge. The second case is the complete opposite of the first case. In the second case, according to boundary condition, as distance *x* increases, height *h* also increases, but the increase rate of free water surface for negative slope is faster than positive slope of impervious boundary. The boundary condition in the third case is set as the heights of pressure heads of both channels are the same. Therefore, for the positive slope the height of free surface of water increase is parallel to the impervious incline boundary.

## 5. Conclusions

The present mathematical model has referenced the method of Hantush and Cruz [8] and Zamani [10] because in their approach the equation of upper flow line has a directional relationship to the average depth of free water surface with respect to the impervious boundary. Equation (14) is the solution of the one-dimensional flow rate of infiltrate water in incline porous media at any distance *x* and height *h*. Looking at all the cases, it can be concluded that the level of free surface of water table can be measured at different distances.

## References

[1] Patel K., Mehta M., Patel T. (2012). A series solution of moisture content in vertical groundwater flow through unsaturated heterogeneous porous media. *International eJournal of Mathematics and Engineering*.

[2] Swaroop A., Mehta M. N. A solution to the problem of one dimensional flow in unsaturated porous media taking Finite Element Approach.

[3] Philips R. (1970). *Advances in Hydro Science*.

[4] Verma A., Mishra S. (1973). A similarity solution of a unidimensional vertical groundwater recharge through porous media. *Revue Roumaine des Sciences Techniques Série de Mécanique Appliquée*.

[5] Mehta M. N. A singular perturbation solution of one-dimensional flow in unsaturated porous media with small diffusivity coefficient.

[6] Bear J. (1979). *The Hydraulics of Groundwater*.

[7] Todd D. K., Mays L. W. (2005). *Groundwater Hydrology*.

[8] Hantush M. M., Cruz J. (1999). Hydrogeologic foundations in support of ecosystem restoration. *United States Enviromental Protection Agency*.

[9] Bear J., Alexander H. (2010). *Modeling Groundwater Flow and Contaminant Transport Dynamics of Fluids in Porous Media*.

[10] Zamani M. (2012). An analysis on groundwater flow in inclined porous media. *International Journal of Science and Advanced Technology*.

